# Efficient green light-emitting diodes based on quasi-two-dimensional composition and phase engineered perovskite with surface passivation

**DOI:** 10.1038/s41467-018-02978-7

**Published:** 2018-02-08

**Authors:** Xiaolei Yang, Xingwang Zhang, Jinxiang Deng, Zema Chu, Qi Jiang, Junhua Meng, Pengyang Wang, Liuqi Zhang, Zhigang Yin, Jingbi You

**Affiliations:** 1 Key Laboratory of Semiconductor Materials Science, Institute of Semiconductors, Chinese Academy of Sciences, Beijing, 100083 China; 2 College of Applied Sciences, Beijing University of Technology, Beijing, 100124 China; 3 College of Materials Science and Opto-electronic Technology, University of Chinese Academy of Sciences, Beijing, 100049 China

## Abstract

Perovskite light-emitting diodes (LEDs) are attracting great attention due to their efficient and narrow emission. Quasi-two-dimensional perovskites with Ruddlesden–Popper-type layered structures can enlarge exciton binding energy and confine charge carriers and are considered good candidate materials for efficient LEDs. However, these materials usually contain a mixture of phases and the phase impurity could cause low emission efficiency. In addition, converting three-dimensional into quasi-two-dimensional perovskite introduces more defects on the surface or at the grain boundaries due to the reduction of crystal sizes. Both factors limit the emission efficiency of LEDs. Here, firstly, through composition and phase engineering, optimal quasi-two-dimensional perovskites are selected. Secondly, surface passivation is carried out by coating organic small molecule trioctylphosphine oxide on the perovskite thin film surface. Accordingly, green LEDs based on quasi-two-dimensional perovskite reach a current efficiency of 62.4 cd A^−1^ and external quantum efficiency of 14.36%.

## Abstract (web summary)

Solution-processable halide perovskites have high luminous efficiency and excellent chemical tunability, making them ideal candidates for light-emitting diodes. Here Yang et al. achieve high external quantum efficiency of 14% in the devices by fine-tuning the phase and passivating the surface defects.

## Introduction

Solution-processed halide perovskite semiconductor materials show good photoluminescence quantum yields (PLQYs)^1–7^, which motivates applications in displays and lighting^8^. The perovskite light-emitting diodes (PeLEDs) have been reported with moderate electroluminescence (EL) efficiencies as well^9–15^. Their external quantum efficiencies (EQEs) have reached about 10% for near-infrared^13–15^ and also in green emission^11,12,15^. Hybrid organic–inorganic perovskites are better known for their free charge carriers with long diffusion length, which is good for solar cells. However, opposite properties are desired when they are used as emitting materials in LEDs^11,16–18^. The idea is to spatially confine charges with the insulating layers, and thus the exciton dissociation is suppressed and the radiative recombination can be enhanced in the perovskite films. Several efficient methods have been adopted, including polymer composite^19^, nanocrystal pinning^11^ and quantum dots^3–7^.

The exciton binding energy is enlarged in the two-dimensional (2D) perovskite and thus the emission efficiency can be enhanced^20,21^. In the early reports, 2D perovskite materials were used as emitting layers, while electroluminescence can only be observed at liquid nitrogen temperature^20^. Recently, quasi-two-dimensional (quasi-2D) perovskites have been proposed by doping 2D perovskites into three-dimensional (3D) perovskite structures. The initial idea was to enhance the humidity stability of perovskite solar cells^22–24^. More recently, it was found that multiple quantum wells could be assembled in these quasi-2D perovskites^13,14^, which can facilitate the formation of exciton and reduce the possibility of exciton dissociation. This makes the quasi-2D perovskite an efficient luminescent material^13–15,25^. The working mechanism of quasi-2D PeLEDs has been fully discussed elsewhere^13,14^. Electrons and holes injected from large bandgap phases (small *n*-phase) can rapidly transfer into small bandgap phases (large *n*-phase), then electrons and holes recombine in large *n*-phase and emit as photons. The EQEs of quasi-2D perovskite LEDs have achieved around 10%^13,14^ and highest EQE of 11.7% in near-infrared region was obtained^14^. Comparing to organic or inorganic LED counterparts^26,27^, there is still room for improvement in the EQE.

It is known that quasi-2D perovskite materials usually contain a mixture of phases and the phase impurity could lead to low emission efficiency, and thus fine control of the composition/phases is critical for efficient emission. In addition, solution processed thin films of quasi-2D perovskite show reduced crystal size compared to those of 3D perovskite^13,15^. This could increase the concentration of the defects and traps on the film surface and grain boundaries, which will act as non-radiative recombination centers and thus decrease the emission efficiency. Surface passivation has been proved an efficient way for reducing the defects in perovskite solar cells^28–30^.

In this article, PEA_2_(FAPbBr_3_)_*n*-1_PbBr_4_ (*n* = 1, 2, …, ∞) is investigated as the emitting layer for quasi-2D perovskite LEDs, where PEA and FA are phenylethylammonium (C_6_H_5_C_2_H_4_NH_3_^+^) and formamidinium (HC(NH_2_)_2_^+^), respectively. Firstly, we optimize the films phase engineering/phase engineering. We find that the perovskite with *n* = 3 composition shows decent phases and the obtained films show the highest photoluminescence (PL) yield. Secondly, we present surface passivation of quasi-2D perovskite films by trioctylphosphine oxide (TOPO) treatment^31^. Accordingly, the PeLEDs based on PEA_2_(FAPbBr_3_)_*n*-1_PbBr_4_ with *n* = 3 composition show green emission with current efficiency (CE) of 62.4 cd A^−1^ and EQE of 14.36%, which is the highest efficiency for PeLEDs reported so far.

## Results

### Properties of quasi-2D perovskite with different compositions

**Fig. 1:**
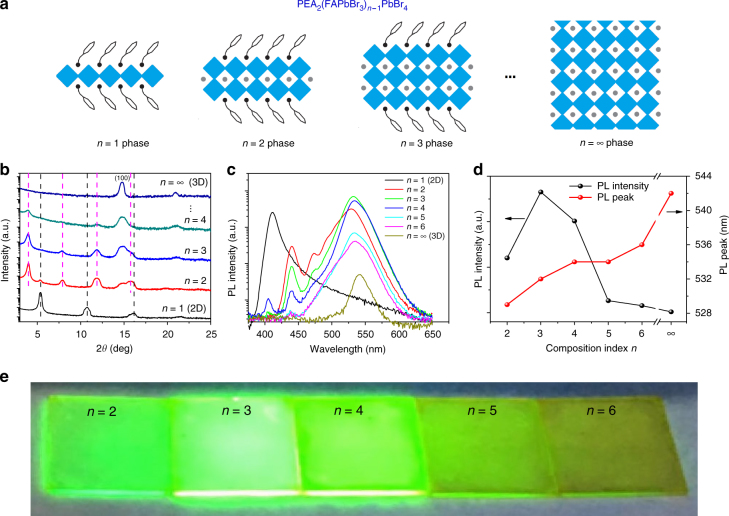
Properties of perovskite films with different compositions. **a** Scheme of PEA_2_(FAPbBr_3_)_*n*-1_PbBr_4_ phases. The black part is PEA, the blue square is PbBr_6_-octahedron and the gray dot is FA. **b** X-ray diffraction patterns of PEA_2_(FAPbBr_3_)_*n*-1_PbBr_4_ films with different *n*-compositions. Diffraction patterns of *n* = 1 phase (black vertical lines), *n* = 2 phase (pink vertical lines) and *n* = ∞ phase (marked with (100)) were labeled. The analysis details can be found in the Supplementary Table 2. **c** Photoluminescence of PEA_2_(FAPbBr_3_)_*n*-1_PbBr_4_ with different *n*-compositions. **d** Photoluminescence intensity and peak of PEA_2_(FAPbBr_3_)_*n*-1_PbBr_4_ films with different compositions with the data collected from **c**. **e** Photoluminescence image of PEA_2_(FAPbBr_3_)_*n*-1_PbBr_4_ films with different compositions under ultraviolet lamp excitation. Figure 1a adapted from ref. 13 (copyright 2016 Macmillan Publishers)

The schematics of *n*-phase PEA_2_(FAPbBr_3_)_*n*-1_PbBr_4_ are drawn in Fig.1a, illustrating that the *n*-phase unit cell contains *n*-1 sheets of PbBr_6_-octahedra and two PEA tiers. The perovskite films are prepared by spin-coating a precursor solution using a crystal-pinning process^11,32^. Their compositions are controlled by the ratio PEABr:FABr:PbBr_2_ (Supplementary Table 1), and compositions *n* ranging from 2 to 6 are discussed. Previous results suggested that the quasi-2D perovskite films are not a single phase but contain a variety of *n*-phases^13,14^. For example, if the precursor solution with *n* = 2 composition is spin-coated, the generated films might include *n *= 2 phase but could also contain other phases such as *n *= 1 or *n* ≥ 3. To avoid misunderstanding, we distinguish the composition and phase by *n*-composition and *n*-phase, respectively. Methylammonium chloride (MACl) is also added into the precursor to improve the morphology and enhance the emission efficiency of the formed films (Supplementary Figs. 1, 2 and 12), but most MACl escape from the film during annealing^33^. The role of MACl has been well investigated in solar cells^33,34^. First, the MACl can slow down perovskite crystallization and improve film morphology. Second, the incorporation of a very small amount of elemental Cl could passivate the grain boundary, and then enhance the emission efficiency (Supplementary Fig. 2).

X-ray diffraction (XRD) measurements are carried out to determine the accurate phases in our obtained quasi-2D perovskite films. As shown in Fig. 1b, all the perovskite films with *n* ≥ 2 compositions show diffraction peaks at 14.8°, which is the same as the (100) diffraction patterns of the 3D perovskite FAPbBr_3_ (refs. ^7,35^). However, the peaks become much more broadened, indicating that the diffraction peak could be from a series of PEA_2_(FAPbBr_3_)_*n*-1_PbBr_4_ phases with large *n* (*n*→∞) or 3D FAPbBr_3_ with nano-size grains (Supplementary Fig.3)^15^. This result shows that all the quasi-2D perovskite films always contain large *n*-phases (*n*→∞) or 3D perovskite regardless of the composition of the precursor. Atomic force microscopy (AFM) images confirm the reduction of the crystal size during the transformation of 3D perovskite into quasi-2D perovskites (Supplementary Fig.3).

In addition to the diffraction peak at 14.8°, a series of Bragg reflections at low angles (2*θ* < 14.8°) are observed for these quasi-2D perovskites (Fig.1b). This indicates that the PEA group with large size is incorporated, and the size of unit cells is enlarged compared with 3D perovskite^14,25,36^. According to the analysis shown in Supplementary Table 2 and the unit cells of *n*-phases shown in Fig.1a, it is found that the diffraction peaks (2*θ* < 14.8°) are attributed to the *n* = 1 and *n* = 2 phases (diffraction patterns from *n* ≥ 3 phases are not observed here). Specifically, for* n* = 1 composition, only the *n *= 1 phase exists; for *n* = 2 composition, the *n* = 1 and* n* = 2 phases are both presented in the films. It is unexpected that diffraction peaks of the *n* = 1 phase is absent in the *n* = 3 composition, while obvious diffraction peaks from the *n* = 2 phase are observed. For *n* ≥ 4 compositions (*n* > 4 compositions are not shown here due to very weak small *n*-phases diffractions), the diffraction peaks from small *n*-phases become weak or absent.

The photoluminescence (PL) results of the quasi-2D perovskite films with different compositions are shown in Fig. 1c, and the 2D (*n* = 1) and 3D (*n* = ∞) perovskite films are also included for comparison. The 2D perovskite shows an emission at 412 nm (3.01 eV) and the long tail^21,37^ at a longer wavelength, which could be due to the self-trapped exciton emission^21^ or the disordered structure. The 3D perovskite showed a band edge emission peak at 542 nm (2.29 eV)^38^. All quasi-2D perovskite films have two main emission peaks: one in the green region (approximately 532 nm, approximately 2.33 eV) and another in the blue region (at 440 nm, 2.82 eV). Comparing the PL with the absorption spectra (Fig.1c and Supplementary Fig. 4), it can be found that the stronger green emission at approximately 532 nm comes from large *n*-phases (*n*→∞) or 3D perovskite, and the weaker blue emission at 440 nm comes from the *n* = 2 phase. The emission phases we find are nearly almost consistent with the XRD results. There are some exceptions: for the *n *= 2 composition, we have not observed the *n* = 1 phase emission, while the *n* = 4 composition shows an emission at 405 nm from the 2D perovskite phase (*n *= 1), and the reason for this is not clear at present.

The relative PL intensity and emission peak wavelength in the green emission from the quasi-2D perovskites and 3D perovskite are plotted in Fig. 1d. It is found that the *n* = 3 composition film shows the brightest green emission. The significant blue-shift of the green emission from quasi-2D perovskites is observed compared with the 3D perovskite. In addition, a smaller *n*-composition showed a larger blue-shift, indicating that the blue-shift is due to the quantum confinement effect. The emission images of these quasi-2D perovskite films under ultraviolet excitation (Fig. 1e) also confirms that the* n* = 3 composition shows the best PL.

Compared with 3D perovskites, significant enhancement of the PL is observed for quasi-2D perovskites (Fig.1c). This is due to the quantum confinement effect via the formation of quantum well structures between large bandgap phases (smaller *n*-phases) and small bandgap phases (*n*→∞ phase or 3D perovskite)^13,14^. The different emissions from various compositions observed in Fig.1c could be explained by the competition between confinement phase (small *n*-phase) and emission phase (large *n*-phase, *n*→∞ phase). Here, we use *n* = 2, 3, 4 compositions as examples. According to XRD results shown in Fig.1b, the *n* = 2 confinement phase and 3D emission phase dominate in *n* = 2 and *n* = 4 composition, respectively, and both of these will cause weak green emission. While the amount of *n* = 2 phase and 3D phase are adequate in the *n* = 3 composition, this could be the reason of the highest PL of *n* = 3 composition. The PLQY of the *n* = 3 shows a value as high as 57.3% under low density excitation (excitation wavelength of 400 nm, power density of 3.5 mW cm^-2^). The quantum well of *n* = 3 composition is mainly formed by the *n *= 2 and* n*→∞ phases (Supplementary Fig. 6).

### Passivation of quasi-2D perovskite

**Fig. 2:**
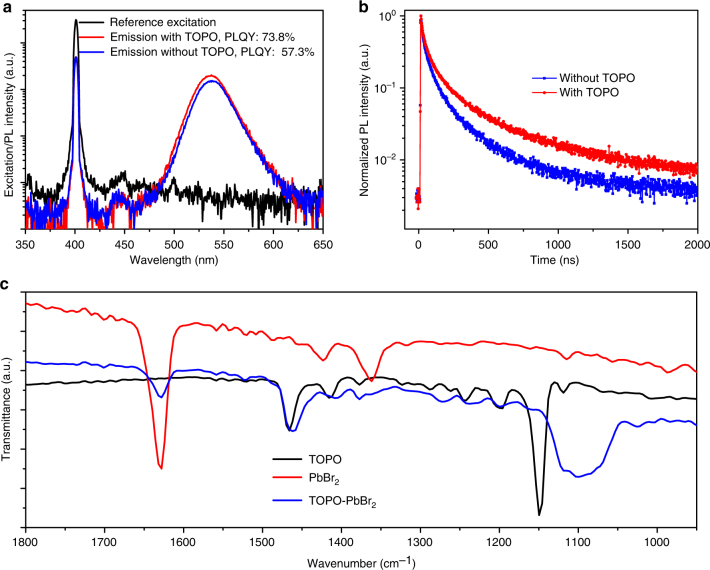
Passivation effect of TOPO on the perovskite with *n* = 3 composition. **a** Photoluminescence quantum yield (PLQY) of the PEA_2_(FAPbBr_3_)_*n*-1_PbBr_4_ (*n* = 3 composition) perovskite films with and without TOPO passivation. **b** Time-resolved photoluminescence (TRPL) of the PEA_2_(FAPbBr_3_)_*n*-1_PbBr_4_ (*n* = 3 composition) films with and without TOPO passivation layer. **c** Fourier transform infrared (FTIR) spectroscopy measurement for TOPO, PbBr_2_ and TOPO-PbBr_2_ films prepared on silicon wafers

Keeping in mind that the *n* = 3 composition of PEA_2_(FAPbBr_3_)_*n*-1_PbBr_4_ perovskite could be a good candidate for an emitting material, we further improve its emission by surface passivation. An organic small molecule TOPO is spin-coated onto the perovskite surface to form a thin passivation layer^31^. It is found that the PLQY of the *n* = 3 composition film was significantly increased from 57.3% to 73.8% after coating with the TOPO layer (Fig. 2a), confirming the passivation effect of the TOPO layer. The average fluorescence lifetime of the *n* = 3 composition film is also increased from 0.17 μs to 0.36 μs after TOPO treatment (Fig. 2b). Although a small red-shift in PL spectra has been observed for the TOPO-treated 3D perovskite film previously^31^, no obvious modulation of PL due to TOPO treatment is observed in this study (Supplementary Fig. 7).

The passivation of halide perovskites is ascribed to the chemical interaction between molecular ligands and incomplete PbI_6_-octahedra in previous study^39^. To confirm this, Fourier transform infrared (FTIR) spectroscopy measurements were carried out, and the result is shown in Fig. 2c. An absorption peak located at 1150 cm^−1^ is observed for TOPO, which corresponds to P=O bond stretching vibrations^40^. This P=O bond absorption peak shifts to approximately 1100 cm^−1^ in the film comprised of TOPO and PbBr_2_, indicating that the bonding between perovskite and TOPO could be formed^41,42^.

### Device structure and performance

**Fig. 3:**
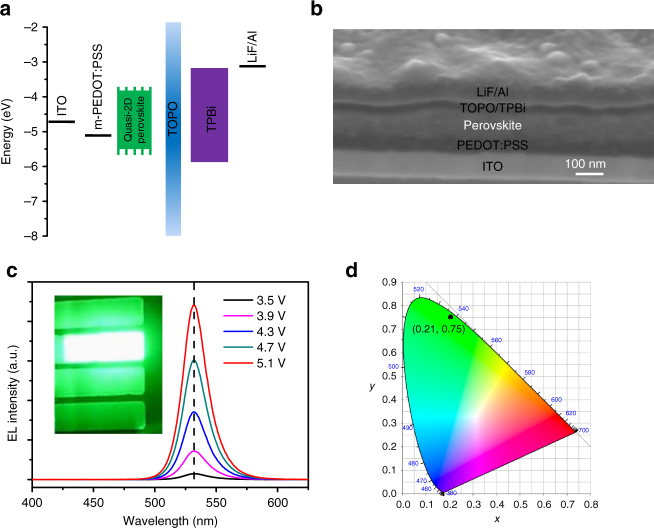
Perovskite light-emitting diodes structure and electroluminescence. **a** Band alignment of each function layer in the devices. **b** Cross-section scanning electron microscopy (SEM) image of the device, the scale bar is 100 nm. **c** Typical electroluminescence (EL) spectra of PEA_2_(FAPbBr_3_)_*n*-1_PbBr_4_ (*n* = 3 composition) based PeLEDs under different voltage bias. Inset shows the electroluminescence image of PeLEDs. **d** The corresponding Commission Internationale de l’Eclairage (CIE) coordinate of typical PeLEDs based on PEA_2_(FAPbBr_3_)_*n*-1_PbBr_4_ (*n* = 3 composition)

We configure PeLEDs as the structure glass/ITO/PEDOT:PSS/PEA_2_(FAPbBr_3_)_*n*-1_PbBr_4_/TPBi/LiF/Al. Modified PEDOT:PSS (m-PEDOT:PSS) is used as the hole injection layer (HIL), TPBi/LiF as the electron injection layer (EIL) and the quasi-2D perovskites as the emitting layer. The band alignment for all function layers is shown in Fig. 3a, and TOPO shown in the figure acts as a thin passivation layer on the perovskite film. Br-based perovskites usually exhibit a deeper valence band (approximately −6.0 eV, Supplementary Fig.5) than that of PEDOT:PSS (approximately −5.2 eV). We doped PSS-Na into PEDOT:PSS to increase the work function^43^, which can improve hole injection as well as device performance (Supplementary Fig.13). The cross-section scanning electron microscopy (SEM) image of the completed device is shown in Fig. 3b, and a clear sandwich device structure is observed. The thicknesses of m-PEDOT:PSS (HIL), the perovskite layer and TPBi (EIL) are about 50, 110 and 30 nm, respectively. The typical EL spectra of the *n* = 3 composition-based PeLEDs under different voltage bias are shown in Fig. 3c. Although the perovskite films exhibit a mixture of many phases with different bandgaps, only single green EL is observed, even in logarithmic scale (Supplementary Fig. 8), which is different from the PL. It can be estimated that the charge density injected by EL (10^17^ s^−1^ cm^−2^) is two orders higher than that excited by PL (10^15^ s^−1^ cm^−2^) under injection/excitation of 5.1 V, 30 mA cm^−2^ and 1 mW cm^−2^, 340 nm excitation, respectively, while the blue emission is still not observed in EL. This could be due to the different working mechanism between EL and PL. For the PL, the photo-excited charges are driven from a larger bandgap phase to a smaller bandgap phase by their conduction/valence band energy difference. However, for EL, the driving force is not only the energy difference, but also the applied external electric field. As a result, most charges will be injected and recombined in the smallest band gap phases. This could be the reason that only green EL is observed even under high charge density injection. The EL peak is located at 532 nm, and the full width at half maximum is about 23 nm. The emission of our PeLEDs exhibited a good color purity (95%) in the green region, with Commission Internationale de l’Eclairage (CIE) chromaticity coordinate at (0.21, 0.75) (Fig. 3d).

**Fig. 4:**
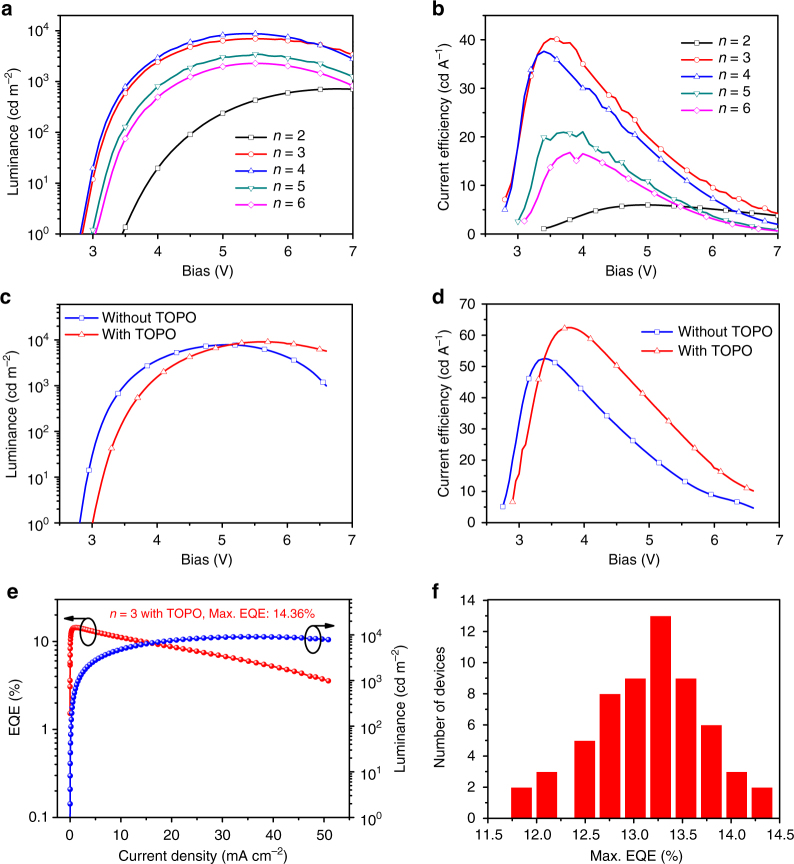
Device performance of perovskite LEDs with different compositions and surface passivation. **a** Luminance–voltage (L-V) curves of PEA_2_(FAPbBr_3_)_*n*-1_PbBr_4_ with different compositions. **b** Current efficiency–voltage (CE-V) curves of PEA_2_(FAPbBr_3_)_*n*-1_PbBr_4_ with different compositions. **c** Luminance–voltage (L-V) curves of PEA_2_(FAPbBr_3_)_*n*-1_PbBr_4_ (*n* = 3 composition) devices with and without TOPO layer. **d** Current efficiency–voltage (CE-V) curves of PEA_2_(FAPbBr_3_)_*n*-1_PbBr_4_ (*n* = 3 composition) devices with and without TOPO layer. **e** External quantum efficiency (EQE) of the champion device of PEA_2_(FAPbBr_3_)_*n*-1_PbBr_4_ (*n* = 3 composition) with TOPO passivation layer. **f** Histogram of maximum EQEs measured from 60 devices

We characterize the PeLEDs with different compositions (Fig. 4a, b and Supplementary Fig. 9). It is found that the *n* = 2 composition showed the lowest injection current (Supplementary Fig. 9), which could be due to the poor transport properties of 2D phase. Correspondingly, the brightness and CE of *n* = 2 composition-based devices are both the lowest (Fig. 4a, b). The compositions of *n* = 3 and *n* = 4 based PeLEDs show the maximum brightness of about 7000 cd m^−2^ and 8700 cd m^−2^, respectively, while *n* = 3 composition shows a higher CE (40.2 cd A^−1^) than* n* = 4 composition (37.6 cd A^−1^). For the *n* = 5 and *n* = 6 compositions, consistent with their PL efficiency (Fig.1c, e), their EL also could not compare with that of *n* = 3 composition. The detailed device performances for various compositions are summarized in Table 1.

**Table 1:** Device performance based on quasi-two-dimensional perovskites PEA_2_(FAPbBr_3_)_*n*-1_PbBr_4_

Compositions	*L*_max_ (cd m^−2^)	Current efficiency (cd A^−1^)	Maximum EQE (%)
*n* = 2	717	5.98	1.54
*n* = 3	6973	40.20	9.29
*n *= 3 (best)	7829	52.51	12.12
*n* = 4	8779	37.61	8.45
*n* = 5	3452	20.95	4.82
*n* = 6	2281	16.77	4.00
*n *= 3 and TOPO	9120	62.43	14.36

We next discuss the role of surface passivation in performance improvement for *n* = 3 composition. It is found that the turn-on voltage increases and the injection current decreases after introducing a TOPO layer (Supplementary Fig.10). This is due to the insulating property of TOPO and indicates that the TOPO is successfully coated on the perovskite surface. Figure 4c shows the brightness curve of the PeLEDs with and without TOPO. The maximum brightnesses of the PeLEDs with and without passivation are at the same level, while the devices with passivation showed higher CE (Fig. 4d). The best performing device without TOPO passivation shows a CE of 52.5 cd A^−1^, while TOPO-treated devices show a CE as high as 62.4 cd A^−1^, and the power efficiency also increased from 49.4 lm W^−1^ to 53.3 lm W^−1^ (Supplementary Fig.11). Accordingly, a high EQE of 14.36% is obtained (Fig. 4e). We also find that our devices show good reproducibility. The EQE histogram for 60 devices from five batches are presented, showing an average EQE of 13.1% with a low relative standard deviation of 4.4% (Fig. 4f).

## Discussion

Device stability is still a critical and common issue in PeLEDs. The stability of our PeLEDs with encapsulation was tested under a constant injection current in dry air (Supplementary Fig. 14). The luminance and CE increased at the beginning for several minutes, which could be due to trap filling^15^ that results in the improvement of electrical contacts. After that, the device performance gradually decreased, and finally the devices work for approximately 120 min. We also noticed that larger injection current induces faster degradation, which could be due to heat accumulation or ion movements. Our device showed the almost same level stability of I-related perovskite with the stability in several 100 min^15^. Compared with the Br-related perovskite with several 10 min stability in previous reports^15,44^, our devices showed a little bit improvement.

Further improvement of PeLED stability is necessary, and we assume that there are several issues leading to PeLED instability. One major issue could be ion migration under voltage bias, and another issue could be the phase stability of perovskite materials under heating or moist conditions. Several strategies could be adopted to improve PeLED stability such as finding stable perovskite-emitting materials^19,45^, incorporating suitable groups to avoid or suppress the ion movements^12,19^, seeking effective ligands^17,46^ to bond with the grain surface and also the film surface to reduce Joule heating induced by non-radiative recombination and finding stable charge injection layers^47^.

In summary, by composition/phase engineering, we find that a quasi-2D perovskite of PEA_2_(FAPbBr_3_)_*n*-1_PbBr_4_ with *n* = 3 composition showed good PL. We also introduce surface passivation to reduce the non-radiative recombination on the perovskite surface or grain boundaries. Combining composition/phase engineering and surface passivation, high performance of quasi-2D PeLEDs with a CE of 62.4 cd A^-1^ and an external quantum efficiency of 14.36% is achieved.

## Methods

### Materials

The modified PEDOT:PSS (m-PEDOT:PSS) solution is a mix of normal PEDOT:PSS (AI 4083) aqueous solution and 100 mg ml^−1^ PSS-Na (Sigma Aldrich) aqueous solution by a volume ratio of 6:5. TOPO, PbBr_2_, dimethyl sulfoxide (DMSO) and chlorobenzene (CB) were purchased from Sigma Aldrich. 2,2’,2”-(1,3,5-Benzinetriyl)- tris(1-phenyl-1-H-benzimidazole) (TPBi) and formamidinium bromide (FABr) and methylammonium chloride (MACl) were purchased from Xi’an Polymer Light Technology Corp. Phenylethylammonium bromide (PEABr) was purchased from Dyesol (now Greatcell Solar).

### Device fabrication

The indium tin oxide (ITO)-coated glass substrates were sequentially cleaned in detergent, distilled water, acetone and isopropanol by sonication and used as anode. The cleaned substrates were ultraviolet ozone treated for 15 min to make the surface hydrophilic, then m-PEDOT:PSS (or normal PEDOT:PSS) aqueous solution was spin-coated at 9000 rpm (or 2500 rpm) for 40 s and baked at 160 °C for 15 min in ambient air. Thereafter, the substrates were transferred into a nitrogen-filled glove box, and the 0.6 mol l^−1^ (Pb^2+^concentration) perovskite solution in DMSO was spin-coated onto the m-PEDOT:PSS (or normal PEDOT:PSS) films at 3000 rpm for 2 min, and after spin coating for 40 s, 100 µl of CB was poured onto the film for pinning the perovskite crystallization, followed by annealing on a hot plate at 90 °C for 1 h. The perovskite precursor solution was prepared according to Supplementary Table 1. For surface passivation, 2 mg ml^−1^ TOPO in CB was dripped onto the perovskite film for a 1 min to let TOPO passivate the film surface enough and then spin-coated at 7000 rpm for 1 min. Finally, the fabrication of PeLEDs was completed by depositing TPBi, LiF (less than 1 nm) and Al electrode layer by layer through a shadow mask in a high vacuum thermal evaporator. The device area was 0.108 cm^2^ as defined by the overlapping area of the ITO and Al electrode. The thickness of ITO and Al are 130 nm and 60 nm, respectively.

### Material and device characterizations

XRD measurements were performed with a Rigaku D/max 2500H equipment with a conventional Cu target X-ray tube (Cu K-alpha, *λ* = 1.5418 Å) set to 40 kV and 200 mA. Steady-state PL spectra and time-resolved fluorescence spectra (TRPL) of the perovskite films were measured at room temperature in the ambient air using a fluorescent spectrophotometer (HORIBA JY Nanolog-TCSPC). The excitation wavelength for PL and TRPL are both 340 nm, which were provided by 450 W xenon lamp and a pulsed diode laser (NanoLed-340), respectively. The PLQY of the perovskite films was measured using an Edinburgh FLS920 equipment with a 450 W xenon lamp as excitation source. The following settings were applied for PLQY measurements: excitation wavelength of 400 nm; bandpass values of 3.00 and 0.35 nm for the excitation and emission slits, respectively; step increments of 0.5 nm and integration time of 0.3 s per data point. We measured the light intensity with a laser power meter and the estimated light power density incident on the samples was about 3.5 mW cm^−2^. The ultraviolet–visible absorbance spectra were recorded on an ultraviolet–visible spectrophotometer (Cary 5000, Varian). The FTIR spectra were recorded on an infrared spectrophotometer (Excalibur 3100, Varian) in a general mode that did not use attenuated total reflection component. AFM images of the perovskite films were collected in non-contact mode (Bruker FASTSCANBIO). The cross-sectional image of PeLEDs was made on a scanning electron/focused ion beam double-beam equipment (FEI Nova200 NanoLab) operated at 2 kV. Ultraviolet photoelectron spectroscopy (UPS) spectra were collected on a Thermo Scientific ESCALab250Xi equipment with an applied bias of −10 V. He I ultraviolet radiation source (21.22 eV) was used. The helium pressure in the analysis chamber during measurement was about 2E−8 mbar. The film samples over the ITO layer had a conductive connection with an Au sample, so the Fermi level value of the film samples is equal to that of Au sample. The work function *ϕ* (that is the Fermi level absolute value of the free film) of the test films can be calculated from following equation: *hν *− *ϕ = E*_Fermi_ – *E*_cutoff_, where *E*_Fermi_ and *E*_cutoff_ are respectively the value of Fermi level position and the steep edge position in the UPS spectrum of the test film, *hν* = 21.22 eV, *E*_Fermi_ is 21.02 eV for Au. Two Keithley 2400 source meter units linked to a calibrated silicon photodiode were used to measure the current–voltage–brightness characteristics. The measurement system was carefully calibrated by a Hamamatsu C9920-02G equipment and a 100 mm integrating sphere of Enli Technology using our perovskite LEDs. Lambertian profile was used in the calculation of EQE. All the device characterization tests of perovskite LEDs were carried out at room temperature in the ambient air for the un-encapsulated devices, except that operational stability test was carried in dry air for the encapsulated devices.

### Data availability

The data that support the findings of this study are available from the corresponding author upon reasonable request.

## Electronic supplementary material

Supplementary Information(PDF 2240 kb)

## References

[1] Deschler F (2014). High photoluminescence efficiency and optically pumped lasing in solution-processed mixed halide perovskite semiconductors. J. Phys. Chem. Lett..

[2] Xing G (2014). Low-temperature solution-processed wavelength-tunable perovskites for lasing. Nat. Mater..

[3] Protesescu L (2015). Nanocrystals of cesium lead halide perovskites (CsPbX_3_, X=Cl, Br, and I): novel optoelectronic materials showing bright emission with wide color gamut. Nano Lett..

[4] Zhang F (2015). Brightly luminescent and color-tunable colloidal CH_3_NH_3_PbX_3_ (X=Br, I, Cl) quantum dots: potential alternatives for display technology. ACS Nano.

[5] Song J (2015). Quantum dot light-emitting diodes based on inorganic perovskite cesium lead halides (CsPbX_3_). Adv. Mater..

[6] Protesescu L (2016). Monodisperse formamidinium lead bromide nanocrystals with bright and stable green photoluminescence. J. Am. Chem. Soc..

[7] Levchuk I (2017). Brightly luminescent and color-tunable formamidinium lead halide perovskite FAPbX_3_ (X=Cl, Br, I) colloidal nanocrystals. Nano Lett..

[8] Kovalenko MV (2017). Properties and potential optoelectronic applications of lead halide perovskite nanocrystals. Science.

[9] Tan ZK (2014). Bright light-emitting diodes based on organometal halide perovskite. Nat. Nanotech..

[10] Wang J (2015). Interfacial control toward efficient and low-voltage perovskite light-emitting diodes. Adv. Mater..

[11] Cho H (2015). Overcoming the electroluminescence efficiency limitations of perovskite light-emitting diodes. Science.

[12] Zhang L (2017). Ultra-bright and highly efficient inorganic based perovskite light-emitting diodes. Nat. Commun..

[13] Yuan MJ (2016). Perovskite energy funnels for efficient light-emitting diodes. Nat. Nanotech..

[14] Wang N (2016). Perovskite light-emitting diodes based on solution-processed self-organized multiple quantum wells. Nat. Photon..

[15] Xiao ZG (2017). Efficient perovskite light-emitting diodes featuring nanometre-sized crystallites. Nat. Photon..

[16] Sutherland BR, Sargent EH (2016). Perovskite photonic sources. Nat. Photon..

[17] Veldhuis SA (2016). Perovskite materials for light-emitting diodes and lasers. Adv. Mater..

[18] Kim YH, Cho H, Lee TW (2016). Metal halide perovskite light emitters. Proc. Natl. Acad. Sci. USA.

[19] Ling Y (2016). Enhanced optical and electrical properties of polymer-assisted all-inorganic perovskites for light-emitting diodes. Adv. Mater..

[20] Era M (1995). Electroluminescent device using two dimensional semiconductor (C_6_H_5_C_2_H_4_NH_3_)_2_PbI_4_ as an emitter. Synth. Met..

[21] Dou L (2015). Atomically thin two-dimensional organic-inorganic hybrid perovskites. Science.

[22] Smith IC (2014). A layered hybrid perovskite solar-cell absorber with enhance moisture stability. Angew. Chem. Int. Ed..

[23] Tsai H (2016). High-efficiency two-dimensional Ruddlesden-Popper perovskite solar cells. Nature.

[24] Quan LN (2016). Ligand-stabilized reduced-dimensionality perovskites. J. Am. Chem. Soc..

[25] Byun J (2016). Efficient visible quasi-2D perovskite light-emitting diodes. Adv. Mater..

[26] Reineke S (2009). White organic light-emitting diodes with fluorescent tube efficiency. Nature.

[27] Pimputkar S (2009). Prospects for LED lighting. Nat. Photon..

[28] Noel NK (2014). Enhanced photoluminescence and solar cell performance via Lewis base passivation of organic-inorganic lead halide perovskites. ACS Nano.

[29] Wang F (2016). Phenylalkylamine passivation of organolead halide perovskites enabling high-efficiency and air-stable photovoltaic cells. Adv. Mater..

[30] deQuilettes DW (2015). Impact of microstructure on local carrier lifetime in perovskite solar cells. Science.

[31] deQuilettes DW (2016). Photoluminescence lifetimes exceeding 8 μs and quantum yields exceeding 30% in hybrid perovskite thin films by ligand passivation. ACS Energy Lett..

[32] Jeon NJ (2014). Solvent engineering for high-performance inorganic–organic hybrid perovskite solar cells. Nat. Mater..

[33] You J (2014). Low-temperature solution-processed perovskite solar cells with high efficiency and flexibility. ACS Nano.

[34] Stranks SD (2013). Electron-hole diffusion lengths exceeding 1 micrometer in an organometal trihalide perovskite absorber. Science.

[35] Meng L (2017). Pure formamidinium-based perovskite light-emitting diodes with high efficiency and low driving voltage. Adv. Mater..

[36] Quan LN (2017). Tailoring the energy landscape in quasi-2D halide perovskites enables efficient green-light emission. Nano. Lett..

[37] Liang D (2016). Color-pure violet-light-emitting diodes based on layered lead halide perovskite nanoplates. ACS Nano.

[38] Arora N (2016). Photovoltaic and amplified spontaneous emission studies of high-quality formamidinium lead bromide perovskite films. Adv. Funct. Mater..

[39] Bi DQ (2016). Polymer-templated nucleation and crystal growth of perovskite films for solar cells with efficiency greater than 21%. Nat. Energy.

[40] Bayou N (2010). Elaboration and characterisation of a plasticized cellulose triacetate membrane containing trioctylphosphine oxyde (TOPO): application to the transport of uranium and molybdenum ions. C. R. Chim..

[41] Söderlind F (2005). Synthesis and characterisation of Gd_2_O_3_ nanocrystals functionalised by organic acids. J. Colloid Interface Sci..

[42] Chang SM, Lo PH, Chang CT (2009). Photocatalytic behavior of TOPO-capped TiO_2_ nanocrystals for degradation of endocrine disrupting chemicals. Appl. Catal. B Environ..

[43] Zuo CT, Ding LM (2016). Modified PEDOT layer makes a 1.52 V V_OC_ for perovskite/PCBM solar cells. Adv. Energy Mater..

[44] Zhang X (2016). Enhancing the brightness of cesium lead halide perovskite nanocrystal based green light-emitting devices through the interface engineering with perfluorinated ionomers. Nano. Lett..

[45] Kulbak M, Cahen D, Hodes G (2015). How important is the organic part of lead halide perovskite photovoltaic cells? Efficient CsPbBr3 cells. J. Phys. Chem. Lett..

[46] Li J (2017). 50-Fold EQE improvement up to 6.27% of solution-processed all-inorganic perovskite CsPbBr_3_ QLEDs via surface ligand density control. Adv. Mater..

[47] You J (2016). Improved air stability of perovskite solar cells *via* solution-processed metal oxide transport layers. Nat. Nanotech..

