# Author Correction: Efficient green light-emitting diodes based on quasi-two-dimensional composition and phase engineered perovskite with surface passivation

**DOI:** 10.1038/s41467-018-03702-1

**Published:** 2018-03-16

**Authors:** Xiaolei Yang, Xingwang Zhang, Jinxiang Deng, Zema Chu, Qi Jiang, Junhua Meng, Pengyang Wang, Liuqi Zhang, Zhigang Yin, Jingbi You

**Affiliations:** 1 Key Laboratory of Semiconductor Materials Science, Institute of Semiconductors, Chinese Academy of Sciences, 100083 Beijing, China; 2 College of Applied Sciences, Beijing University of Technology, 100124 Beijing, China; 3 College of Materials Science and Opto-electronic Technology, University of Chinese Academy of Sciences, 100049 Beijing, China

**Correction to**: *Nature Communications* (2017) 10.1038/s41467-018-02978-7; Article published online: 08 Feb 2018

The original version of this Article omitted an acknowledgement to the source of Fig. 1a. The following has been added to the end of the caption to Fig. 1:

‘Figure 1a adapted from ref. 13 (copyright 2016 Macmillan Publishers)’.

This has been corrected in the PDF and HTML versions of the Article.

