# Supplementary material for: Efficient green light-emitting diodes based on quasi-two-dimensional composition and phase engineered perovskite with surface passivation
Source: Nat Commun. 2018 Feb 8;9:570. doi: 10.1038/s41467-018-02978-7 (PMC5805756; doi:10.1038/s41467-018-02978-7)
Supplement: Supplementary file 1 — Supplementary Information(PDF 2240 kb) [file 41467_2018_2978_MOESM1_ESM.pdf]

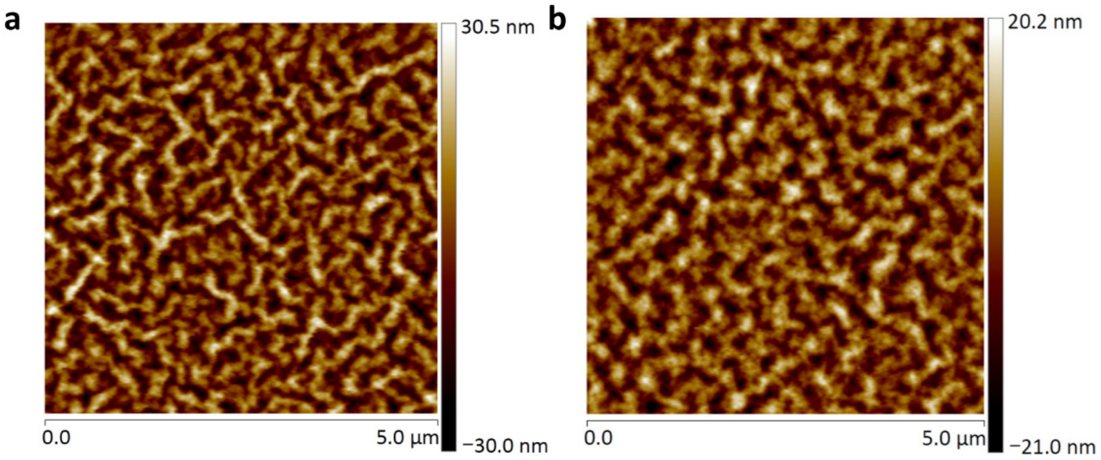

Supplementary Figure 1 | Atomic force microscope (AFM) images of the $PEA_2(FAPbBr_3)_{n-1}PbBr_4$ (n=3 composition) perovskite films. a, Without MACl additive. **b**, With MACl additive.

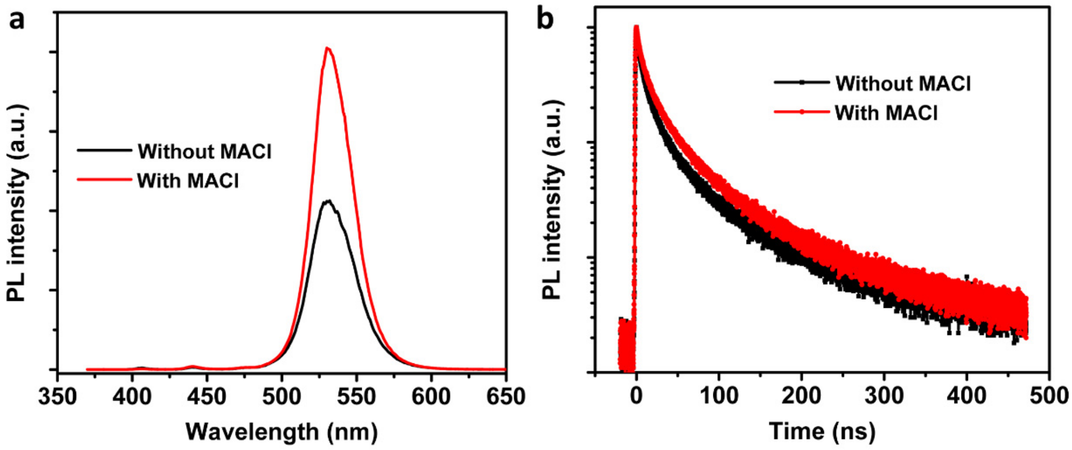

Supplementary Figure 2 | Photoluminescence (PL) of $PEA_2(FAPbBr_3)_{n-1}PbBr_4$ (n=3 composition) films with and without MACl additive. a, Steady-state PL spectra of $PEA_2(FAPbBr_3)_{n-1}PbBr_4$ (n=3 composition) perovskite films with and without MACl additive. **b**, Time resolved photoluminescence (TRPL) spectra of $PEA_2(FAPbBr_3)_{n-1}PbBr_4$ (n=3 composition) perovskite films with and without MACl additive.

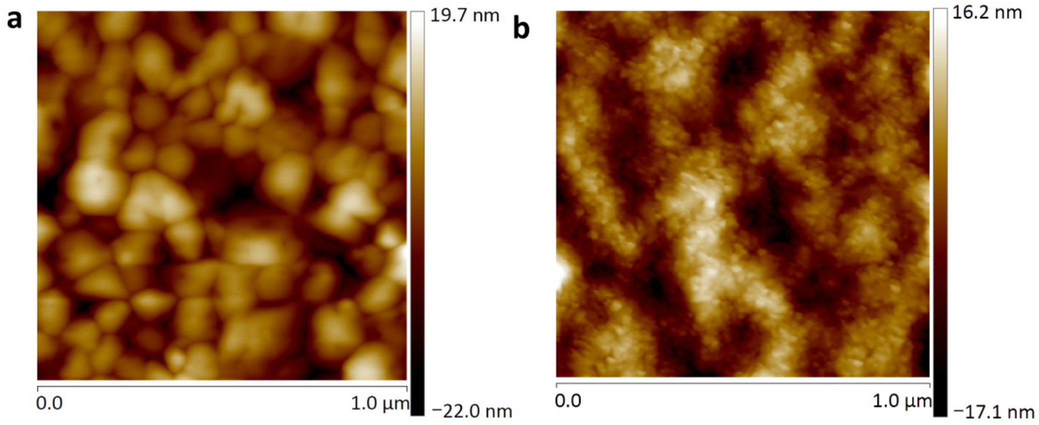

Supplementary Figure 3 | Morphology of perovskite films. **a**, Atomic force microscope (AFM) images of the **t**hree dimensional $FAPbBr_3$. **b,** AFM image of quasi-two dimensional $PEA_2(FAPbBr_3)_{n-1}PbBr_4$ (n=3 composition).

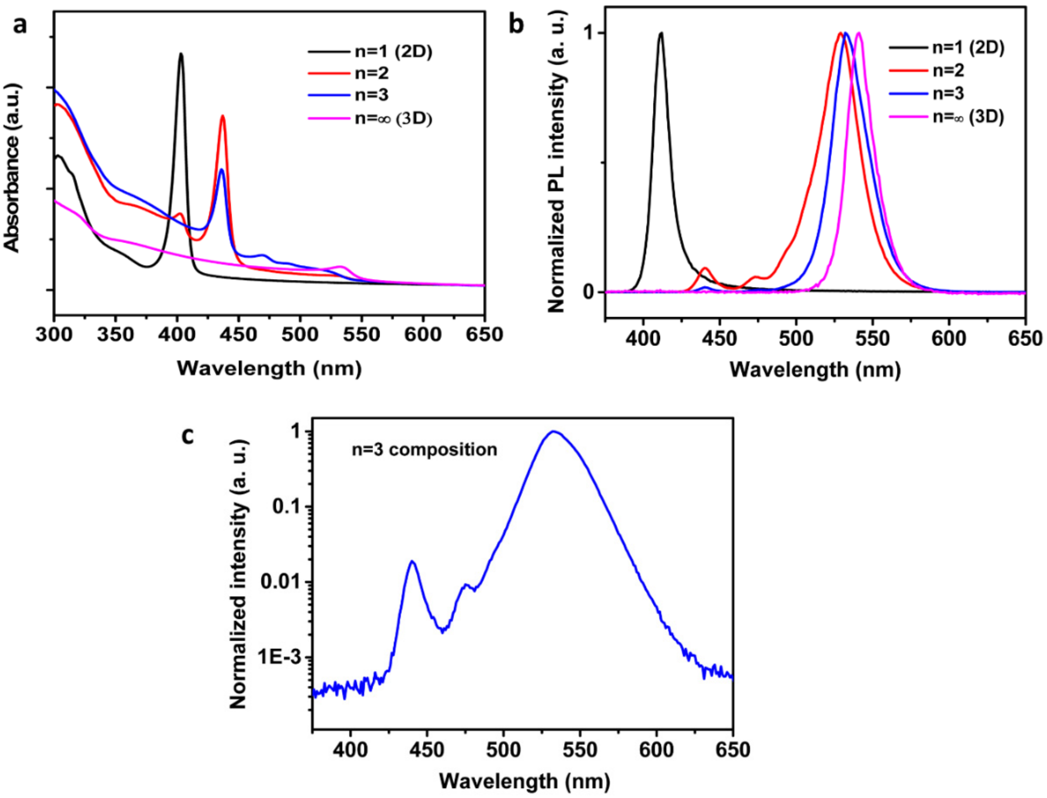

Supplementary Figure 4| Absorption and emission of perovskite with different compositions. a, Absorption of the perovskite films with different compositions. **b**, Normalized photoluminescence of the perovskite films with different compositions. **c**, Photoluminescence of the $PEA_2(FAPbBr_3)_{n-1}PbBr_4$ perovskite film with n=3 composition in logarithm scale.

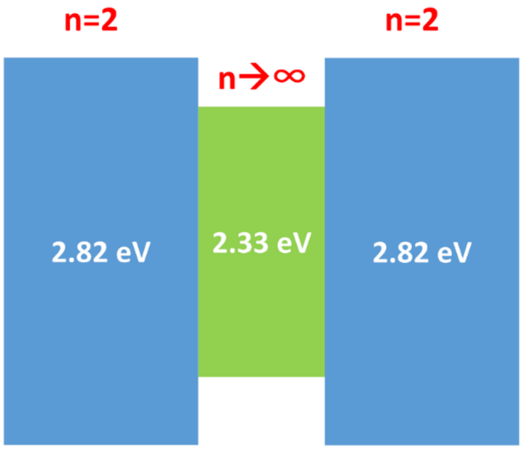

Supplementary Figure 5 | Self-organized quantum wells in the quasi-2D $PEA_2(FAPbBr_3)_{n-1}PbBr_4$ perovskite film with n=3 composition. The main phases in the n=3 composition are n=2 (large band gap, 2.82 eV) and n→∞(small band gap, 2.33 eV) phases, the quantum well structures was formed by these two main phases.

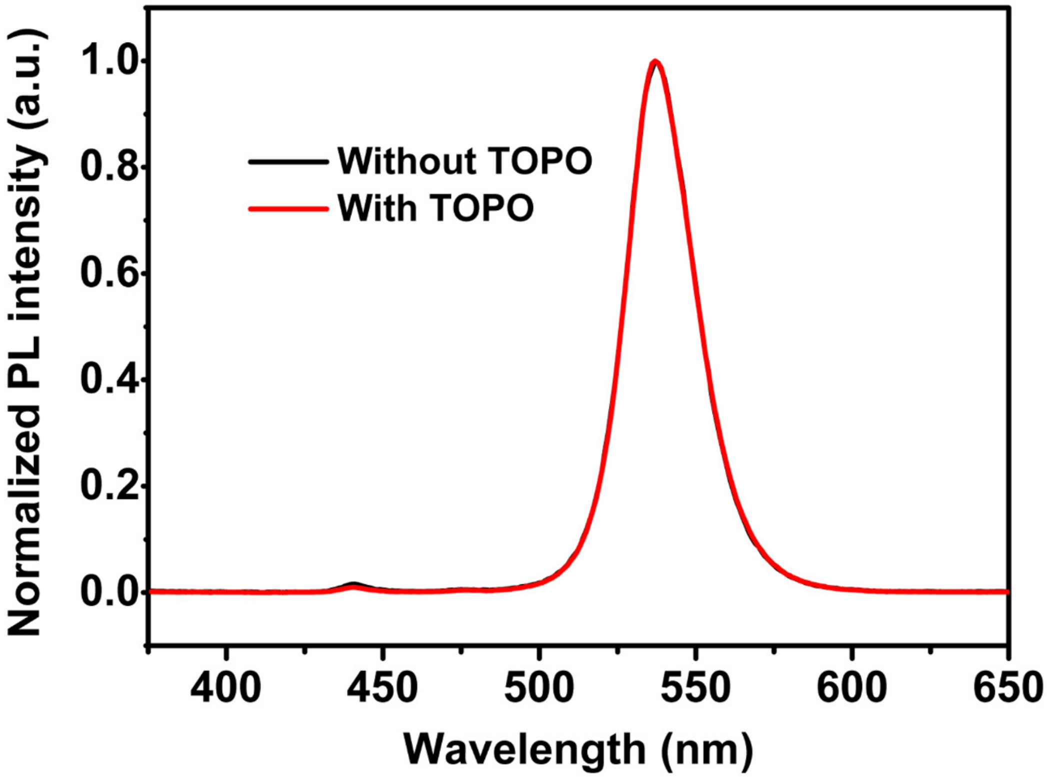

Supplementary Figure 6| Emission properties of perovskite films with and without passivation. Normalized photoluminescence of quasi-two dimensional perovskite $PEA_2(FAPbBr_3)_{n-1}PbBr_4$ (n=3 composition) without and with TOPO passivation layer.

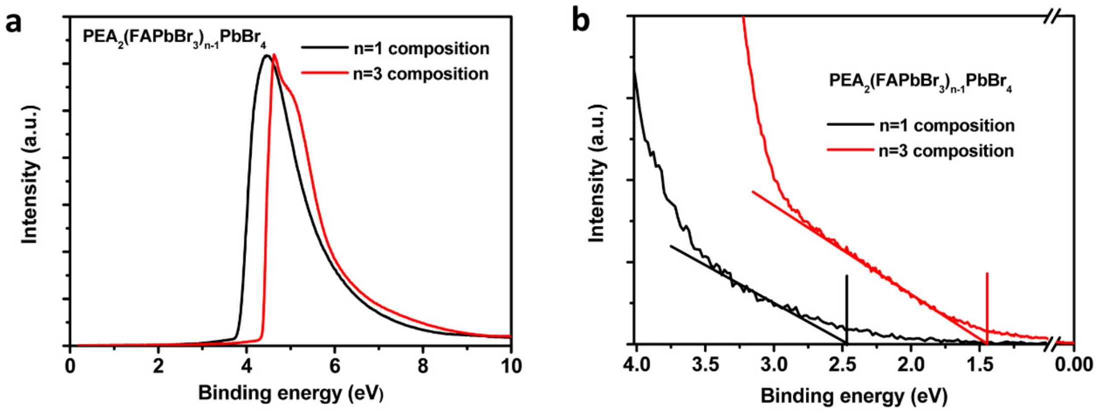

Supplementary Figure 7| Measurement of band structure of perovskite films. a, Ultraviolet photoelectron spectroscopy (UPS) cutoff of $PEA_2(FAPbBr_3)_{n-1}PbBr_4$ perovskite films with n=1 and n=3 composition, respectively. **b**, Valence band spectra of the $PEA_2(FAPbBr_3)_{n-1}PbBr_4$ perovskite films with n=1 and n=3 composition, respectively. It can be calculated that the valence band of the $PEA_2(FAPbBr_3)_{n-1}PbBr_4$ with n=1 and n=3 are located at -6.28 eV and -5.80 eV, respectively.

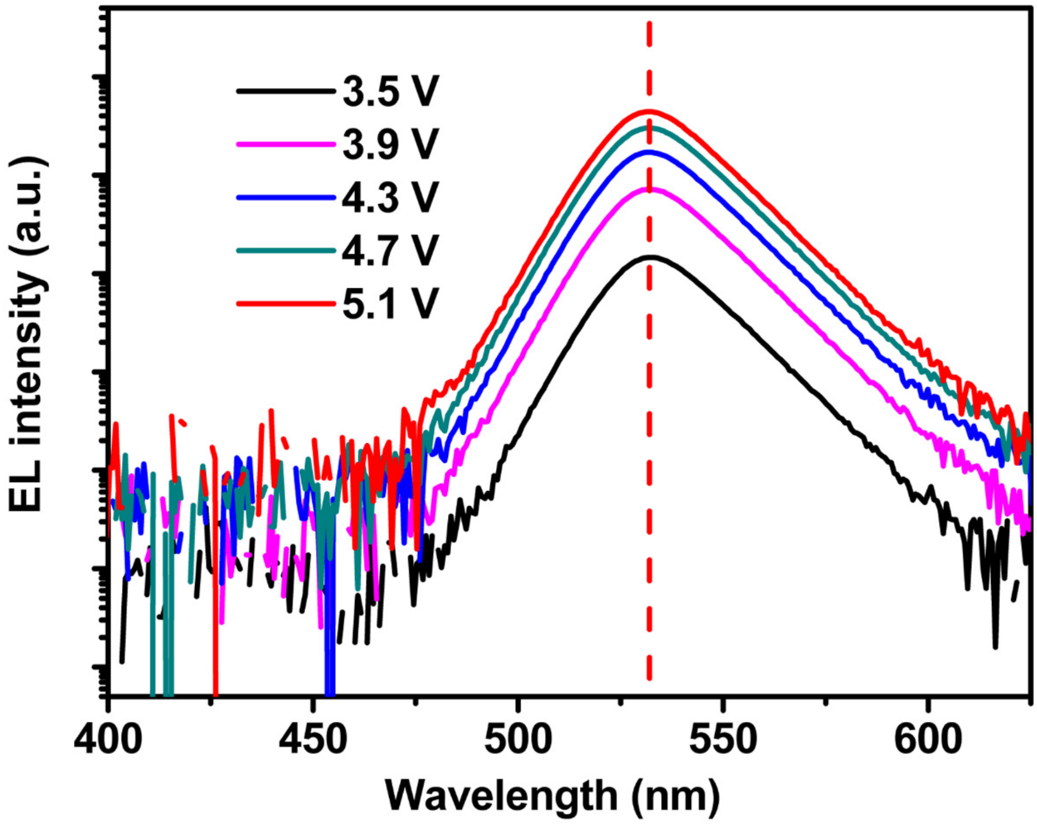

Supplementary Figure 8| Electroluminescence properties. Electroluminescence (logarithmic scale) of quasi-two dimensional perovskite $PEA_2(FAPbBr_3)_{n-1}PbBr_4$ (n=3 composition) under different applied voltage. This is the logarithmic scale of Fig. 3c.

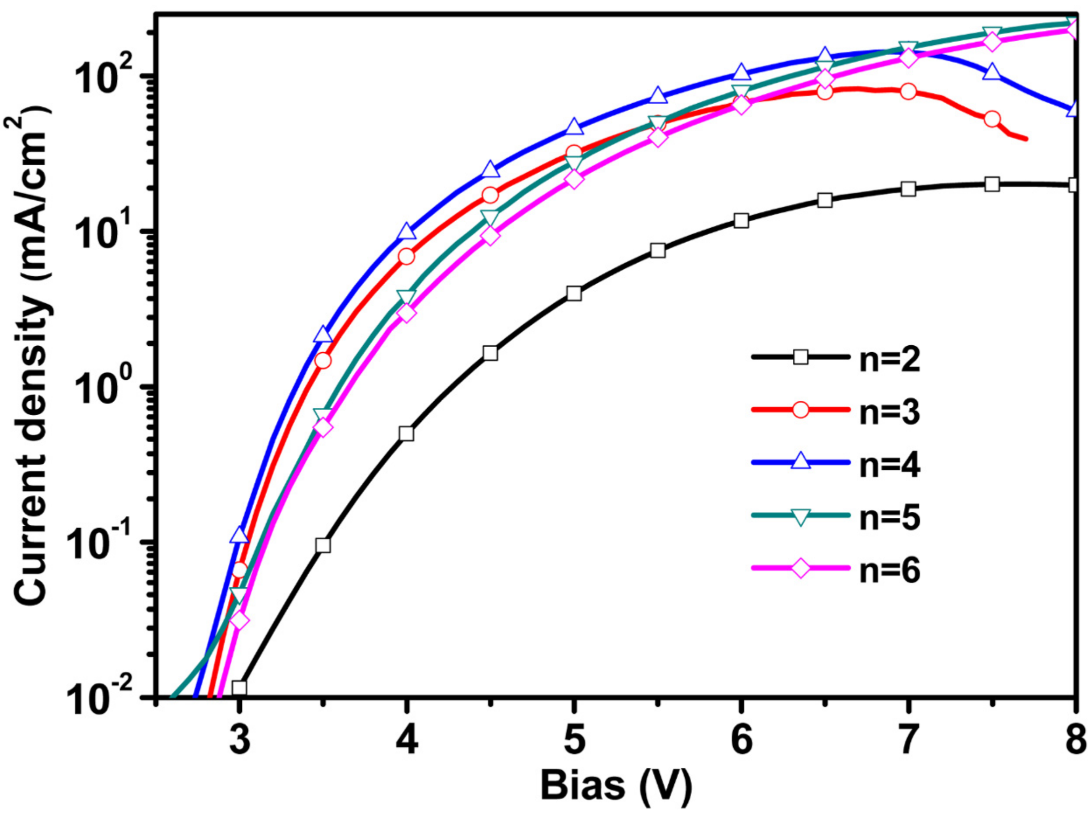

Supplementary Figure 9| Injection properties of the devices with different compositions. Current density versus voltage bias (J-V) curves for LED devices based on different $PEA_2(FAPbBr_3)_{n-1}PbBr_4$ (n=2, 3, 4, 5, 6) compositions.

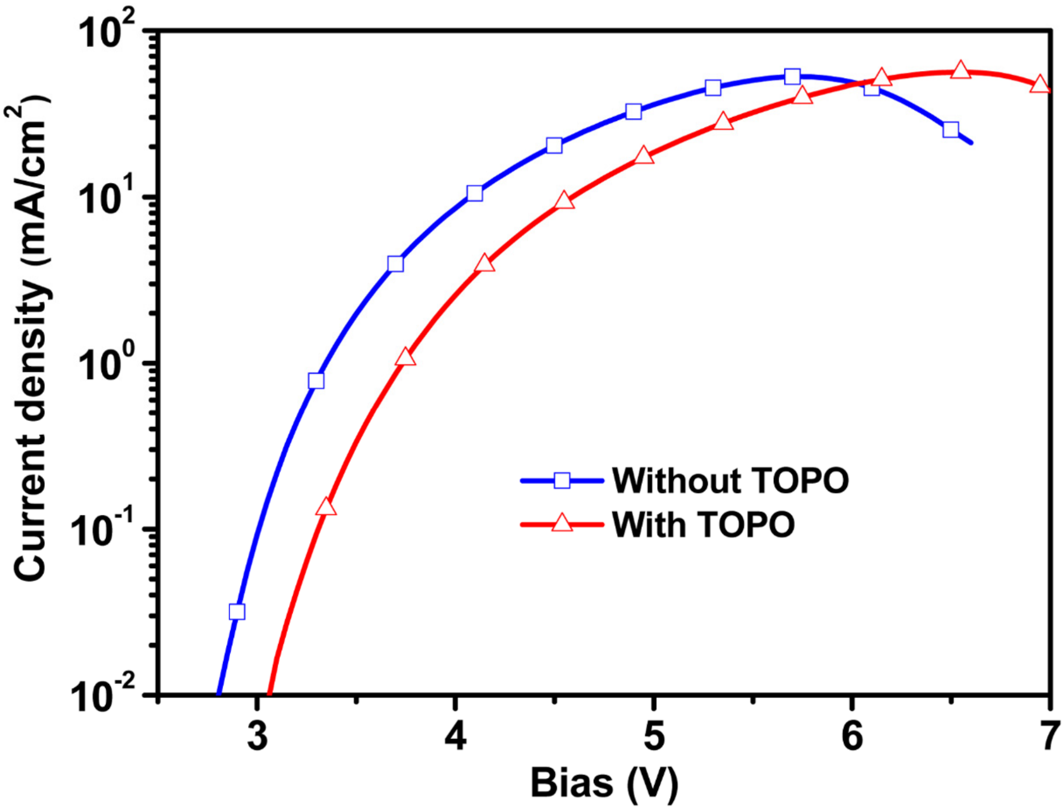

Supplementary Figure 10| Injection properties of the devices with and without passivation. Current density versus applied voltage bias for the $PEA_2(FAPbBr_3)_{n-1}PbBr_4$ (n=3 composition) based perovskite light emitting diodes with and without trioctylphosphine oxide (TOPO) passivation layer.

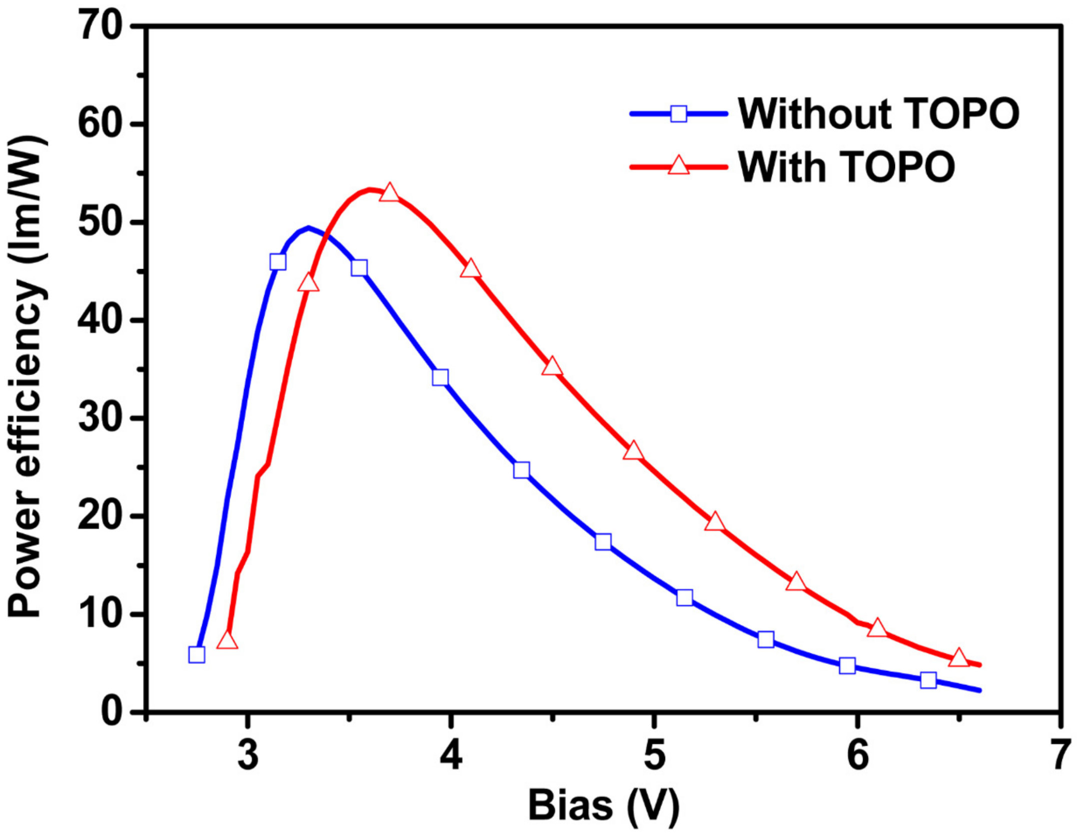

Supplementary Figure 11 | Power efficiency of the devices with and without passivation.

Power efficiency of the $PEA_2(FAPbBr_3)_{n-1}PbBr_4$ (n=3 composition) based perovskite light-emitting diodes with and without trioctylphosphine oxide (TOPO) passivation layer.

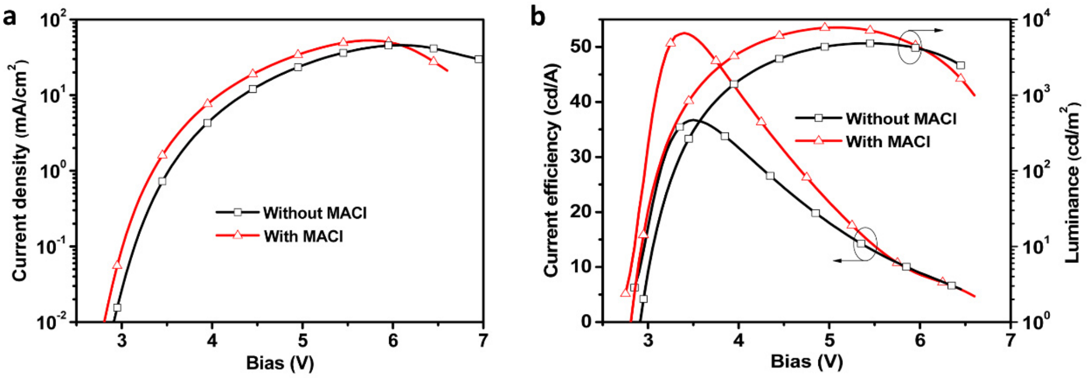

Supplementary Figure 12 | Devices performance of perovskite light-emitting diodes with and without methylammonium chloride (MACl) additive. a, Current density –bias curve of $PEA_2(FAPbBr_3)_{n-1}PbBr_4$ (n=3 composition) light-emitting diodes with and without MACl additive. b, Luminance-bias and current efficiency-bias curve of $PEA_2(FAPbBr_3)_{n-1}PbBr_4$ (n=3 composition) light-emitting diodes with and without MACl additive. Trioctylphosphine oxide (TOPO) passivation layer was not used in these LED devices.

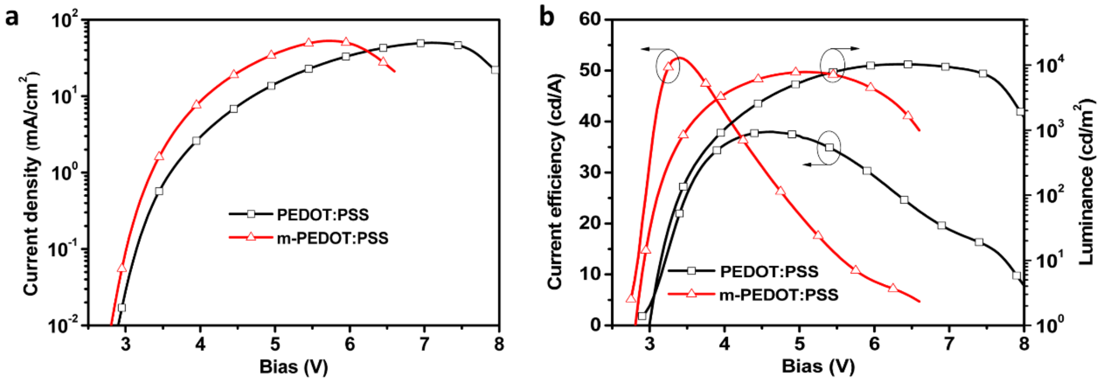

Supplementary Figure 13 | The effects of hole transport layer on device performance. **a**, Current density–bias curve of $PEA_2(FAPbBr_3)_{n-1}PbBr_4$ (n=3 composition) light-emitting diodes using normal and modified PEDOT:PSS. **b**, Luminance-bias and Current efficiency-bias curve of $PEA_2(FAPbBr_3)_{n-1}PbBr_4$ (n=3 composition) light-emitting diodes using normal and modified PEDOT:PSS. Trioctylphosphine oxide (TOPO) passivation layer was not used in these LED devices.

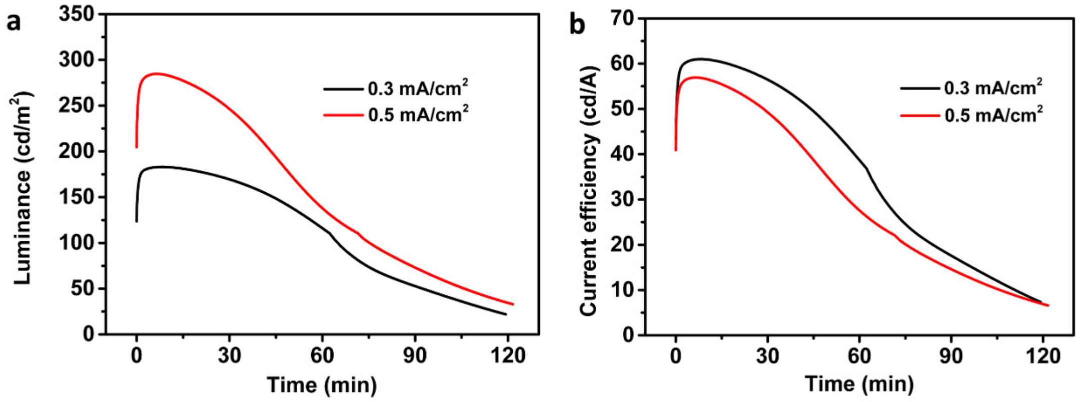

Supplementary Figure 14 | Stability of the encapsulated devices. a, Luminance versus operational time **b**, Current efficiency (CE) versus operational time. The current density of 0.3 mA/cm^2 and 0.5 mA/cm^2 have been used for testing the device stability. The devices were encapsulated by the UV-epoxy and was tested in dry air environment. The devices with the structure of ITO/PEDOT:PSS/$PEA_2(FAPbBr_3)_{n-1}PbBr_4$ (n=3 composition)/TOPO/TPBi/LiF/Al.

Supplementary Table 1| The precursor solution preparation for $PEA_2(FAPbBr_3)_{n-1}PbBr_4$ with different compositions. PEABr, FABr and MACl are phenylethylammonium bromide, formamidinium bromide and methylammonium chloride, respectively.

Composition	$PbBr_2$ (/mL)	FABr (/mL)	PEABr (/mL)	MACl (/mL)
n=2	0.6mmol (220.2mg)	0.3mmol (37.5mg)	0.6mmol (121.2mg)	0.06mmol (4.05mg)
n=3	0.6mmol (220.2mg)	0.4mmol (50.0mg)	0.4mmol (80.8mg)	0.06mmol (4.05mg)
n=4	0.6mmol (220.2mg)	0.45mmol (56.2mg)	0.3mmol (60.6mg)	0.06mmol (4.05mg)
n=5	0.6mmol (220.2mg)	0.48mmol (60.0mg)	0.24mmol (48.5mg)	0.06mmol (4.05mg)
n=6	0.6mmol (220.2mg)	0.5mmol (62.5mg)	0.2mmol (40.4mg)	0.06mmol (4.05mg)

Supplementary Table 2| Analysis of the X-ray diffraction patterns in Fig. 1b. Bragg diffraction equation: 2dsinθ=mλ (m=1, 2, 3···), d is the lattice/unit constant, θ is the diffraction angle, m is the order, and λ=1.54Å for Cu Kα. The lattice constant of 3D (n=∞) perovskite phase is calculated by (100) peak at 14.8° and the layer spacing of the n=1 and n=2 phases is calculated by peaks marked with the black and pink vertical line respectively. The XRD patterns from 2D (n=1) and 3D (n=∞) perovskite are shown in Figure 1b. It can be calculated that the lattice constants of 2D and 3D perovskite are 16.4 Å and 6.0Å, respectively. It can be estimated that the phase of $PEA_2(FAPbBr_3)_{n-1}PbBr_4$ should be 16.4+(n-1)*6.0 Å according to the scheme shown in Fig.1a.

**XRD peak**	**n**	**2θ**	**θ**	**m**	**d (Å)**
(100)	∞	14.76	7.38	1	6.0
pink vertical line	2	3.98	1.99	1	22.2
		7.88	3.94	2	22.4
		11.84	5.92	3	22.4
		15.76	7.88	4	22.5
black vertical line	1	5.36	2.68	1	16.5
		10.78	5.39	2	16.4
		16.16	7.96	3	16.4
